# Community pharmacists’ acceptance of prescribing pre-exposure prophylaxis (PrEP) for human immunodeficiency virus (HIV)

**DOI:** 10.1177/17151635231152218

**Published:** 2023-02-28

**Authors:** Connor Booker, Andrea L Murphy, Jennifer E. Isenor, Tasha D. Ramsey, Alesha J. Smith, Andrea Bishop, Deborah V. Kelly, Lisa Woodill, Greg Richard, Kyle John Wilby

**Affiliations:** College of Pharmacy, Faculty of Health, Dalhousie University, Halifax; College of Pharmacy, Faculty of Health, Dalhousie University, Halifax; College of Pharmacy, Faculty of Health, Dalhousie University, Halifax; College of Pharmacy, Faculty of Health, Dalhousie University, Halifax; Pharmacy Department, Nova Scotia Health Authority, Halifax, Nova Scotia; School of Pharmacy, University of Otago, Dunedin, New Zealand; College of Pharmacy, Faculty of Health, Dalhousie University, Halifax; Nova Scotia College of Pharmacists, Halifax, Nova Scotia; School of Pharmacy, Memorial University of Newfoundland, St. John’s, Newfoundland; Pharmacy Association of Nova Scotia, Halifax; Boyd’s Pharmasave, Halifax, Nova Scotia; College of Pharmacy, Faculty of Health, Dalhousie University, Halifax

## Abstract

**Background:**

Pre-exposure prophylaxis (PrEP) for human immunodeficiency virus (HIV) prevention is highly effective. Pharmacists can increase PrEP accessibility through pharmacist prescribing. This study aimed to determine pharmacists’ acceptance of a pharmacist PrEP prescribing service in Nova Scotia.

**Methods:**

A triangulation mixed methods study consisting of an online survey and qualitative interviews was conducted with Nova Scotia community pharmacists. The survey questionnaire and qualitative interview guide were underpinned by the 7 constructs of the Theoretical Framework of Acceptability (affective attitude, burden, ethicality, opportunity costs, intervention coherence, perceived effectiveness and self-efficacy). Survey data were analyzed descriptively and with ordinal logistic regression to determine associations between variables. Interview transcripts were deductively coded according to the same constructs and then inductively coded to identify themes within each construct.

**Results:**

A total of 214 community pharmacists completed the survey, and 19 completed the interview. Pharmacists were positive about PrEP prescribing in the constructs of affective attitude (improved access), ethicality (benefits communities), intervention coherence (practice alignment) and self-efficacy (role). Pharmacists expressed concerns about burden (increased workload), opportunity costs (time to provide the service) and perceived effectiveness (education/training, public awareness, laboratory test ordering and reimbursement).

**Conclusion:**

A PrEP prescribing service has mixed acceptability to Nova Scotia pharmacists yet represents a model of service delivery to increase PrEP access to underserved populations. Future service development must consider pharmacists’ workload, education and training as well as factors relating to laboratory test ordering and reimbursement.

**Knowledge into Practice**

- Pre-exposure prophylaxis (PrEP) is a critical tool for HIV prevention.
- This study found that pharmacists in Nova Scotia have mixed acceptance of a PrEP prescribing service offered by pharmacists.
- Findings have implications for pharmacists’ scope of practice and improving access to PrEP for underserved populations.

**Mise En Pratique Des Connaissances**

- La prophylaxie préexposition (PrEp) est une stratégie indispensable à la prévention du VIH.
- Cette étude a révélé que l’acceptation des pharmaciens de la Nouvelle-Écosse d’un service de prescription pour la PrEP offert par les pharmaciens est mitigée.
- Les résultats ont des implications pour le champ d’activité des pharmaciens et l’amélioration de l’accès à la PrEP pour les populations mal desservies.

## Introduction

The Canadian government has pledged by 2030 to reduce the number of newly acquired human immunodeficiency virus (HIV) infections to zero.^1^ Pre-exposure prophylaxis (PrEP) with medications is one of the mechanisms to achieve this goal, with 99% efficacy in reducing the risk of acquiring HIV when used consistently.^2-6^ PrEP consists of taking antiretrovirals, which in Canada are a combination of tenofovir disoproxil fumarate plus emtricitabine or tenofovir alafenamide plus emtricitabine, prior to exposure to HIV to prevent infection. Prescribing PrEP also includes laboratory monitoring (e.g., bloodwork) for organ health (e.g., kidney) and testing for several sexually transmitted infections and other blood-borne pathogens.^7^ The use of PrEP is rising nationally. In 2018, there were an estimated 10,000 PrEP users across 8 Canadian provinces, representing a 2000% increase from 2014.^8^ Despite the increase, disparities remain in the uptake of PrEP based on various characteristics (e.g., age, sex, race, geography).^7,8^

Nationally, differences in scopes of practice exist in terms of pharmacist prescribing. In Nova Scotia, there are several types of pharmacist prescribing supported by legislation (prescription renewals, contraception management, shingles, uncomplicated urinary tract infections and for the prevention of Lyme disease).^9^ Internationally, most PrEP prescribing services currently occur as part of collaborative practice agreements with other prescribers and not pharmacists independently prescribing within their scope of practice.^10^ Generally, increasing independent PrEP prescribing by pharmacists may increase accessibility of PrEP for those who are underserved and in need. Pharmacists are accessible and trusted by the public.^11^ Pharmacists embedded within communities can provide a stigma-free environment for those with previous negative experiences within the broader health care system.^12^ Pharmacy-based PrEP services, including prescribing of PrEP medications and laboratory monitoring, could reduce barriers to PrEP access for those at greater risk of HIV exposure, including, but not limited to, sexually and gender diverse (2SLGBTQ+) individuals, heterosexual sero-discordant couples and persons who inject drugs.^1,7^ A recent review article by Kennedy et al. in 2022 showed high patient satisfaction with PrEP provided at pharmacies in the United States and South Africa as well as positive findings for pharmacists’ willingness and general support to provide the service.^13^

Before implementation of pharmacist-led independent PrEP prescribing service in a Canadian context, a comprehensive analysis of pharmacists’ perceptions is required for facilitator and barrier identification. For example, a previous study has shown that pharmacists have concerns regarding increased workload with a PrEP prescribing service and insufficient training to be able to provide the service.^13^ Pharmacists with concerns about workload and their capabilities may be less likely to engage in service delivery. Acceptability of interventions to health care professionals can therefore be an important factor in the uptake and sustainability of interventions and warrants measurement prior to, during and after service implementation. The Theoretical Framework of Acceptability (TFA) for health care interventions consists of 7 constructs (affective attitude, burden, ethicality, intervention coherence, opportunity costs, perceived effectiveness, self-efficacy) of acceptability and can be used to examine health care users’ or providers’ cognitive and emotional responses to an intervention.^14^

To determine the facilitators and barriers to acceptance of a PrEP prescribing service by pharmacists in Nova Scotia, a mixed methods study using an online survey and qualitative individual interviews was conducted.

## Methods

### Study design

This was a triangulation mixed methods study consisting of an anonymous cross-sectional survey followed by semistructured qualitative interviews. This study was approved by the Health Sciences Research Ethics Board at Dalhousie University (2022-6044).

### Participants

Pharmacists licensed to practise direct patient care in Nova Scotia who practise in the community setting were eligible to participate in the survey (*N* = 1128). Pharmacists then self-identified their interest in an interview using a separate linked survey (to preserve anonymity) at the completion of the primary survey.

### Questionnaire and discussion guide development

The questionnaire was underpinned by the TFA.^14^ A literature review identified previous studies that assessed pharmacists’ perceptions of PrEP prescribing. Questionnaire items identified were extracted and organized under each of the theoretical framework constructs and evaluated for relevance. Additional items were generated by investigators and categorized to the TFA. The final questionnaire was piloted with 2 pharmacists who made minor comments for wording clarity. The final questionnaire consisted of 31 items that included 7 demographic questions and 24 Likert-type items related to the TFA (affective attitude = 3, burden = 5, ethicality = 4, intervention coherence = 2, opportunity costs = 3, perceived effectiveness = 2, self-efficacy = 5). Likert-type item choices included *strongly disagree*, *disagree*, *neutral*, *agree* and *strongly agree*. All questionnaire items had the response option of “prefer not to answer.” The interview guide included 7 main questions, with 1 for each of the TFA constructs (Appendix 1). The guide was piloted with 2 interviewees, and responses were included in the analysis. Prompting occurred when the interviewer determined elaboration of responses was warranted.

### Data collection

The survey was deployed using REDCap electronic data capture tools hosted at Dalhousie University from May 22 to July 29, 2022.^15,16^ The survey link was distributed in a weekly newsletter from the Pharmacy Association of Nova Scotia as well as through an email sent to all eligible pharmacists by the Nova Scotia College of Pharmacists. Survey responses were regularly monitored for those interested in participating in an interview. Participants were contacted using convenience sampling (i.e., first-come basis) by 1 of the investigators (C.B.) to obtain informed consent via email and schedule an interview for those who provided consent. No additional eligibility criteria were required for interviews. All interviews occurred virtually using Microsoft Teams and were audiorecorded and transcribed verbatim. The interviewer (C.B.) was trained by the primary investigator (K.J.W.), and 2 interviews were conducted together before C.B. completed all other interviews independently. Interviews were conducted until it was determined that little to no new information was being elicited from participants. All interviewed participants received a $30 gift card for their time. No incentive was provided for survey participants.

### Data analysis

The quantitative and qualitative data were analyzed separately and integrated in the determination and interpretation of results using a triangulation design. Survey data were analyzed descriptively, and all completed and partially completed (participants were allowed to skip demographic questions) were included. Responses for *strongly agree* and *agree*, as well as for *strongly disagree* and *disagree* were combined for reporting purposes into *agree* and *disagree*, respectively. Ordinal logistic regression was used to determine the influence of demographic variables (frequency of prescribing activities, years of practice, pharmacy type and frequency of PrEP dispensing) on the questionnaire item, “I am not interested in prescribing PrEP.” Due to the distribution of responses for frequency of PrEP dispensing, the responses for “never” and “less than monthly” were combined, as well as the responses for “monthly,” “weekly” and “daily” for this analysis. All statistical analyses were conducted with IBM SPSS Statistics verson 28 (IBM, Armonk, NY).

Interview recordings were deductively coded according to the 7 constructs of the TFA.^14^ Transcription and coding occurred within 5 days after the interview. Coding according to the TFA was completed by 1 investigator (C.B.) and checked by another (K.J.W.). The nature of responses was continually reviewed by 2 investigators (C.B., K.J.W.) during this process to assess the need for additional interviews. Two investigators (C.B., K.J.W.) independently reviewed the deductively coded data and performed an inductive thematic analysis to generate themes for each construct.^17^ Transcripts were re-reviewed to search for supporting and refuting evidence in the form of quotes to demonstrate trustworthiness of the data. Results from this process were shared with the larger investigator team to confirm final interpretation of data.

## Results

A total of 214 eligible community pharmacists in Nova Scotia completed the required survey components. Demographic information is presented in Table 1. Most respondents identified as female (60%) and had practised pharmacy for 10 or more years (60%). Most of the pharmacists worked in the central region of Nova Scotia (49%), were staff pharmacists (43%) and were employed by a chain pharmacy (48%). More than half (53%) of respondents reported never having dispensed PrEP, with another 32% reporting that they dispensed PrEP less than once a month. On average, respondents reported that they prescribed for a medication approximately 60 times per month.

**Table 1:** Demographic characteristics of survey respondents

Variable	*n* (%)
Number of pharmacists	
Completed survey	211
Partially completed survey	3
Total	214
Gender	
Female	128 (60.4)
Male	65 (30.7)
Nonbinary	2 (0.9)
Gender fluid	1 (0.5)
Prefer not to answer	18 (8.5)
Region of Nova Scotia	
Central	104 (49.1)
Western	42 (19.8)
Eastern	40 (18.9)
Northern	19 (9)
Prefer not to answer	7 (3.3)
Years practising	
<5	37 (17.5)
5-10	43 (20.3)
10-20	66 (31.1)
>20	62 (29.2)
Prefer not to answer	4 (1.9)
Practice setting	
Chain pharmacy	102 (48.1)
Pharmacy within a large store	48 (22.6)
Independent pharmacy	47 (22.2)
Other	9 (4.2)
Prefer not to answer	6 (2.8)
Primary position	
Staff pharmacist	90 (42.5)
Pharmacy manager	69 (32.5)
Pharmacy owner	22 (10.4)
Relief pharmacist	13 (6.1)
Casual pharmacist	11 (5.2)
Other	2 (0.9)
Prefer not to answer	5 (2.4)
Identifies as part of the 2SLGBTQ+ community	
No	180 (85.3)
Yes	19 (9)
Prefer not to answer	12 (5.7)
Frequency of PrEP dispensing	
Never	113 (53.3)
Less than monthly	68 (32.1)
Monthly	24 (11.3)
Weekly	5 (2.4)
Daily	1 (0.5)
Prefer not to answer	1 (0.5)

Eighty-four survey respondents self-identified, and 19 were interviewed for the study. Each interview was approximately 20 to 30 minutes in length. Interviews were stopped at this point as little new information was being discussed by participants.

Survey results according to the TFA constructs are presented in Table 2 and qualitative findings in Table 3. Logistic regression analysis resulted in no significant findings (data not shown). A description of findings from both analyses, including the identification of themes within each construct, is provided below.

**Table 2:** Nova Scotia pharmacist perceptions of prescribing PrEP obtained from the survey and mapped to the Theoretical Framework of Acceptability constructs.

		*n* (%)			
Perception	Construct	Disagree	Neutral	Agree	Prefer not to answer
Patients would prefer to have pharmacists oversee all aspects (e.g., monitoring bloodwork, prescribing medications, follow-up) of PrEP and its prescribing.	Affective attitude	65 (30.4)	60 (28)	88 (41.1)	1 (0.5)
Prescribing PrEP will have a positive influence on my job satisfaction.	Affective attitude	73 (34.1)	57 (26.6)	84 (39.3)	0
I am not interested in prescribing PrEP.	Affective attitude	105 (49.1)	27 (12.6)	82 (38.3)	0
Providing a PrEP prescribing service would be a burden on my current workload.	Burden	53 (24.8)	31 (14.5)	128 (59.8)	2 (0.9)
My pharmacy team has the capacity to initiate PrEP prescribing as a service to patients.	Burden	91 (42.5)	37 (17.3)	86 (40.2)	0
It would take too much time to train other nonpharmacist staff members regarding their roles and responsibilities with PrEP prescribing services.	Burden	99 (46.3)	50 (23.4)	65 (30.4)	0
It will be difficult for me to make time to learn about PrEP prescribing and monitoring.	Burden	95 (44.4)	30 (14)	89 (41.6)	0
I am worried about taking on the responsibility of managing PrEP.	Burden	77 (40)	36 (16.8)	101 (47.2)	0
Pharmacists should have the right to conscientiously object to prescribing PrEP on religious, moral or personal grounds.	Ethicality	108 (50.5)	41 (19.2)	58 (27.1)	7 (3.3)
I believe providing PrEP to patients will increase risky sexual behaviours.	Ethicality	185 (86.4)	14 (6.5)	12 (5.6)	3 (1.4)
PrEP prescribing by pharmacists will benefit the communities they serve.	Ethicality	16 (7.5)	39 (18.2)	154 (72)	5 (2.3)
PrEP prescribing by pharmacists would increase access for people who normally are unable to access this service.	Ethicality	22 (10.3)	20 (9.3)	167 (78)	5 (2.3)
Pharmacists that prescribe PrEP will be responsible for monitoring required laboratory tests (e.g., HIV, STIs, kidney and liver tests).	Intervention coherence	42 (19.6)	37 (17.3)	130 (60.7)	5 (2.3)
Nonpharmacist staff members can help with nonclinical workflow (e.g., schedule patients organize documentation) for PrEP patients.	Intervention coherence	32 (15)	27 (12.6)	153 (71.5)	2 (0.9)
Prescribing PrEP will interfere with my other services and duties in the pharmacy.	Opportunity cost	64 (29.9)	34 (15.9)	115 (53.7)	1 (0.5)
Providing PrEP prescribing will financially benefit the pharmacy I work at.	Opportunity cost	47 (22)	83 (38.8)	82 (38.3)	2 (0.9)
I would need to hire more staff (e.g., assistant, pharmacist overlap) to help me accommodate PrEP prescribing services in my workflow.	Opportunity cost	58 (27.1)	57 (26.6)	97 (45.3)	2 (0.9)
Pharmacist prescribing of PrEP will address the needs of my patient.	Perceived effectiveness	50 (23.4)	72 (33.6)	89 (41.6)	3 (1.4)
Pharmacist prescribing of PrEP will help to prevent, identify and treat sexually transmitted and blood-borne infections.	Perceived effectiveness	29 (13.6)	47 (22)	135 (63.1)	3 (1.4)
I will be capable of prescribing and monitoring PrEP therapy after training.	Self-efficacy	35 (16.4)	28 (13.1)	146 (68.2)	5 (2.3)
I would be confident with having to break bad news of positive sexual health test results to patients.	Self-efficacy	75 (35)	40 (18.7)	99 (46.3)	0
I am confident in providing sexual health advice to patients in the 2SLGBTQ+ community.	Self-efficacy	65 (30.4)	43 (20.1)	106 (49.5)	0
I am confident in providing patient care services to intravenous drug users.	Self-efficacy	45 (21)	32 (15)	136 (65.6)	1 (0.5)
I am capable of finding resources to refer patients to when I am unable to help patients within my scope of practice.	Self-efficacy	29 (13.6)	37 (17.3)	146 (63.6)	2 (0.9)

Data were collapsed to Negative (strongly disagree, disagree), Neutral and Positive (agree, strongly agree).

**Table 3:** Identified themes, interpretation and supporting evidence from interview data for pharmacists’ prescribing of PrEP as per the Theoretical Framework of Acceptability constructs

Construct	Theme	Description	Representative quotes
Affective attitude	Enthusiasm for new role	Pharmacists were overwhelmingly in favour of providing a pharmacist-managed prescribing program for PrEP in Nova Scotia.	I think it’s an excellent idea. I’m all for it. It’s something that I think [that] pharmacists would really be positioned well to offer this as a service. (Participant 12) I feel confident in it. I definitely feel like it’s something that pharmacists could directly be involved in. And at the end of the day it’s for the benefit of the patient and any partners that they may have. I am completely for it. (Participant 17)
	Importance of improving access	Pharmacists had positive attitudes toward prescribing PrEP as a mechanism to increase access to the medication in Nova Scotia. Access related to availability of prescribers, increased patient satisfaction, as well as convenience of pharmacies.	I think that it would be really important for connecting patients to competent care and care that they feel comfortable accessing, especially considering the shortage of family physicians in Nova Scotia, especially in rural communities. (Participant 6) I think it would be definitely a huge step forward in the profession, but I think most importantly it would improve access for people. I think there’s huge stigma attached to starting PrEP and to remove some of the barriers of booking appointments and going to your doctor or trying to source it online, you could just pop into your community pharmacy and talk to someone that you’ve probably known for 10 plus years about it. I think would be a lot easier for people. (Participant 10)
	Moving towards a clinical role	Pharmacists expressed interest in a pharmacist-managed PrEP prescribing service as it would allow pharmacists to practise at full scope in clinically oriented roles.	I think offering more services is a good fit for pharmacy in general, because it starts to open up and the more services we have the more innovative we are. (Participant 1) I think that those types of clinical services are definitely the route that many pharmacists, including myself, want to take. Getting to use clinical skills more often rather than just sort of your typical run-of-the-mill dispensing services, you know, I think that that’s certainly the direction that pharmacy is going. (Participant 16)
Burden	Concern about increased workload	Pharmacists are concerned that adding a PrEP prescribing service will cause increases to workload, having negative impacts to an already very busy practice.	I think it does definitely add to the workload. We’ve had a lot of additional things put on us during the pandemic especially. So, it definitely will increase the workload. But I think it is something that is necessary and probably can be fit into most pharmacists’ workload without overstraining the whole system. (Participant 7) I think that . . . one of the major issues in community pharmacy at this time is workload, with all the expanded scope services that we are now able to provide. While it’s great and again those are the types of clinical services that most of us are more interested in doing, the design of the current workflow in most community pharmacies just is not optimized to support that. (Participant 16)
	Finding workload solutions	Pharmacists proposed solutions to the perceived increase in workload burden. These solutions included appointment-based scheduling, specifying days of the week for clinics and increasing overlap time between pharmacists.	I work at a busier pharmacy. We are going to have a pharmacist work clinical days on certain days of the week. So, I definitely think it will be easier for our model where we’re going to have a clinical day where we will be doing all different types of clinical aspects. So, I do think it’ll be an easy addition to our workload. (Participant 19) I think at my site, in particular we would be OK with implementing a PrEP prescribing service, because we have gotten quite used to doing appointment-based services. And have built our workflow most days to be able to support that fairly easily. (Participant 2)
Ethicality	Values alignment	Pharmacists believed that PrEP prescribing by pharmacists aligns with their personal values.	I think it aligns very highly [with my values] because I think it is access to very important care for a marginalized and underserved population that has demonstrably worse health outcomes. (Participant 6)
	Contributing to harm reduction	Pharmacists stated PrEP is an important method of harm reduction to decrease the spread of HIV and improve health outcomes.	I can’t see anything that would be an ethical dilemma of giving this to somebody because it’s about harm reduction and prevention, not about encouraging anything. It’s about protecting their health. (Participant 3)
	Putting the patient first	Pharmacists felt that the beliefs of their patients come first and that their own belief systems should not have influence on the decision to provide appropriate care.	Ever since pharmacy school, I’ve always been one that believes in putting your own belief system behind to uphold the profession. And you know, as long as it’s within the patient’s belief system that should be all that matters. (Participant 10)
Intervention coherence	Establishing a PrEP process	Pharmacists were familiar with the steps required for PrEP assessment, prescribing and monitoring.	The way I would envision it would be appointment-based services. The pharmacist would review the patient’s desire for accessing PrEP, why they think they need it, whether they would meet like eligibility criteria or at least gain benefit from it. And then if they met the criteria, they would have a full STI screen and they would need their bloodwork done. So, they need to check their renal function and HIV test. And then obviously discussion around whether it’s daily PrEP or on-demand PrEP. If their bloodwork results are good and then they would have their prescription. (Participant 12)
Opportunity costs	Finding the time	Pharmacists suggested that in order to adequately provide a PrEP prescribing service, they would mainly be giving up time that could be used to perform other pharmacy duties.	The reality is that something would have to give. We can’t continue to add to pharmacists’ plate without something giving. Whether that’s intentional or unintentional, right? So, whether you know, we intentionally limit the other services that we’re able to provide in a specific time frame or whether unintentionally the quality of our other work suffers due to time constraints and workflow issues. (Participant 16)
	Alignment with practice	Many pharmacists believed that there would be no opportunity cost to their practice.	I would see it more so as like a bonus or like a benefit on top of what you’re currently doing. . . . But I can’t see anything that I had to give up in order to provide that service. (Participant 3) No, I mean I think the purpose of the expansion of our scope is always additive rather than a replacement. So no, it would never be in my understanding that something new coming to our pharmacy would have to sacrifice something else. (Participant 10)
Perceived effectiveness	Gaining skills	Pharmacists felt that they would require further education to be able to effectively provide a PrEP prescribing service.	I think that there needs to be an education piece along with it, right? I think that’s the key. I think it’s difficult to just throw it on someone’s plate like, “ Alright, here you go and have at it.” (Participant 12) I think the biggest burden [to effectiveness] is going to be the learning curve and pharmacists getting an understanding of the requirements and knowing about it because at this point we really don’t, not all of us, know much about it or have seen it or see it regularly. (Participant 14)
	Creating public awareness	Public knowledge of the service was deemed as an important characteristic of an effective service.	It’s just kind of getting the word out there. So kind of having different resources to promote these types of [services] would be really important and different at all centres throughout the province. (Participant 19)
	Using a protocol	Pharmacists believed adhering to a set protocol for PrEP prescribing would increase the effectiveness of the service.	We are very good at following the rules. So, when there’s a protocol, when there’s an algorithm, when we are told that we must follow a certain procedure, we are quite good at doing that. So, if we have a protocol for PrEP prescribing, we would be very good at following that and prescribing appropriately. (Participant 15)
	Authority to order lab tests	Pharmacists believed the authority to order lab tests should be operationalized to ensure effectiveness of the service.	The logistics aren’t in place for pharmacists to order bloodwork right now. I know it’s been in the works for a while and we hear that it’s very, very, very close. But until that system is established, there’s no real way for us to monitor safety and efficacy of PrEP. (Participant 6)
	Reimbursement model	Adequate reimbursement was identified as a facilitator to increase the effectiveness of a PrEP prescribing service.	It will need to be tied to appropriate compensation because if that is not there, then I do see it being difficult having uptake because it will take up time and if that time’s not being reimbursed then I could see that being a problem (Participant 15)
Self-efficacy	Seeing myself in the role	Pharmacists overwhelmingly perceived themselves to be future PrEP prescribers.	I could definitely see myself as a PrEP prescriber. (Participant 1) I can absolutely see myself providing that type of service. It is in the area I’m more interested in—the more clinical side of things, rather than the technical or dispensing side of things. (Participant 16)

HIV, human immunodeficiency virus; PrEP, pre-exposure prophylaxis; STI, sexually transmitted infection.

### Construct 1: Affective attitude

The results from the Affective Attitude construct of the survey indicated that approximately half (49%) of the respondents were interested in pharmacists prescribing PrEP, while 38% reported they were not interested. Forty-one percent of pharmacists felt that patients would prefer having pharmacists manage all aspects of PrEP prescribing. Pharmacists were divided on whether PrEP prescribing would positively influence job satisfaction. Three themes were identified from the qualitative analysis for this construct and represented positive attitudes toward PrEP prescribing: “Enthusiasm for new role,” “Importance of improving access” and “Moving towards a clinical role” (Table 3). Themes encompassed positive attitudes related to the positive effects of prescribing PrEP for both the pharmacist and patients.

### Construct 2: Burden

A total of 60% of pharmacists agreed that a PrEP prescribing service would be a burden on their current workload, and 47% indicated that they were worried about the responsibility of managing PrEP for patients. Mixed results were obtained on whether respondents’ pharmacies had the capacity to initiate a PrEP prescribing service and if they thought that it would be difficult for them to make time to learn about PrEP prescribing. Forty-six percent of respondents reported that it would not take too much time to train nonpharmacist staff about their roles in a PrEP prescribing service. The qualitative analysis identified 2 themes relating to workload: “Concern about increased workload” and “Finding workload solutions” (Table 3). One theme captured respondents’ perceptions on how to reduce the perceived workload burden, including appointment-based services, designated clinic days and greater staffing levels as potential solutions.

### Construct 3: Ethicality

Most pharmacists disagreed that PrEP increases risky sexual behaviours (86%) and just over half disagreed that they should have the right to object to prescribing PrEP (51%). Furthermore, pharmacists agreed that prescribing PrEP will increase access to the medication (72%) and benefit their communities (78%). Three themes were identified for this construct through the qualitative analysis: “Values alignment,” “Contributing to harm reduction” and “Putting the patient first.” These themes encompassed pharmacists’ values relating to providing quality care for individual patients, as well as through protecting communities by contributing to harm reduction initiatives.

### Construct 4: Intervention coherence

Most (61%) pharmacists agreed that pharmacists would be responsible for monitoring laboratory tests in a PrEP prescribing service, and 72% agreed that nonpharmacist staff members can help with nonclinical workflow of PrEP prescribing. From the qualitative analysis, 1 theme was identified to support this construct: “Establishing a PrEP process.” Interviewed pharmacists were generally aware of the steps that would be involved in managing a PrEP prescribing service.

### Construct 5: Opportunity costs

About half of pharmacists (54%) felt a PrEP prescribing service would interfere with their other pharmacy duties, with 45% indicating it would require them to hire more staff to accommodate the changes in pharmacy workflow. Pharmacists were divided on whether providing the service would financially benefit their pharmacies. The qualitative analysis identified 2 themes for this construct: “Finding the time” and “Alignment with practice.” Time for other activities was identified as the main concern of interview respondents, while many felt that they would not be required to “give up” anything additional to provide this service.

### Construct 6: Perceived effectiveness

Most pharmacists (63%) agreed that a PrEP prescribing service would help prevent, identify and treat sexually transmitted and blood-borne infections and that a pharmacist-managed PrEP prescribing service would address the needs of their patients (42%). Five themes were identified for this construct from the qualitative analysis: “Gaining skills,” “Creating public awareness,” “Using a protocol,” “Authority to order lab tests” and “Reimbursement model.” For the service to be effective, some pharmacists identified specific education and training needs but were confident in the protocol nature of PrEP prescribing. Pharmacists also indicated that the public would need to be educated to increase awareness of the service, that pharmacists would need to have the authority to order laboratory tests and that the service would need to be remunerated to increase the likelihood of success.

### Construct 7: Self-efficacy

Most pharmacists (68%) were confident that they would be able to perform the tasks required to provide a PrEP prescribing service after proper training. Sixty-three percent of respondents agreed that they would be capable of referring patients when the situation was outside of their scope of practice, and 46% felt confident that they would be able to “break bad news” of a positive sexual health test result to patients. Half (50%) of the respondents reported having confidence in providing sexual health advice to patients in the 2SLGBTQ+ community, while 66% responded they were confident in providing care to people who use injection drugs. The qualitative analysis identified 1 theme: “Seeing myself in the role.” Interviewed pharmacists overwhelmingly perceived themselves as future PrEP prescribers and were positive about the capabilities to provide care to PrEP patients.

## Discussion

This study determined that Nova Scotia pharmacists had mixed acceptance of a PrEP prescribing service by pharmacists, based on the 7 constructs of the TFA.^14^ Pharmacists’ acceptance of PrEP prescribing was especially evident within the constructs of affective attitude, ethicality, intervention coherence and self-efficacy. They believed that prescribing PrEP would increase access to underserved populations and would help pharmacists practise at full scope. More negative results were found within the constructs of burden, opportunity costs and perceived effectiveness, and the qualitative interview data provided greater context to pharmacists’ concerns within these areas.

The primary concern related to burden and the extra workload that a PrEP prescribing service would place on the pharmacy staff. Findings that pharmacists needed to “find the time” for this service within the construct of opportunity costs are common in pharmacy practice research and may be a function of services being added without adequate staffing assessments.^18^ This is also highlighted by the frequency that pharmacists reported prescribing activities (approximately 60 times per month). Although solutions were identified to reduce the impact of extra workload by pharmacists during the interview phase (e.g., appointment-based services, ensuring overlap of pharmacists on duty, clinic days), workload is an important point that must be addressed. This is especially important in the context of the unpredictable and unprecedented workflow challenges created by forces internal and external to pharmacies (e.g., vaccine supply, immunization clinics, staff shortages, public health policy changes) during the COVID-19 pandemic.^19^ However, the protocolized nature of PrEP prescribing was highly valued and deemed to support pharmacists’ abilities to prescribe PrEP and may, in part, help to manage some aspects of workload.

Notably, more than half of survey respondents had never dispensed PrEP as part of their current workload, and only 50% of pharmacists were confident providing sexual health advice to 2SLGBTQ+ patients. Pharmacists also expressed the desire to have education in these areas, which may have influenced their acceptance. While the number of eligible patients who receive PrEP in Nova Scotia is unknown, it is estimated that patient numbers would be lower than other prescribing indications, but this information was not provided to survey respondents. Respondents may have therefore conceptualized a PrEP prescribing service to be similar to other prescribing services offered, such as for contraception or urinary tract infections, in which prescribing may be more common. Lack of prevalence knowledge, limited dispensing experience and perceived deficits in education may all contribute to perceptions of increased workload with a PrEP service and should be addressed.

This study has limitations that should be noted. First, we had approximately 200 survey respondents, which represented 19% of the entire eligible population. Although more responses would have been desired, this sample represented community pharmacists in Nova Scotia with differing attitudes and perceptions toward PrEP prescribing. For the survey and interviews, pharmacists self-selected with no random process for determining participation, thereby potentially introducing selection bias. Pharmacists were allowed to skip questions in the survey, which may have resulted in some bias toward favourable outcomes. However, skipped responses were few (Table 2). Some questions may also be specific to Nova Scotia pharmacists and lack generalizability. Specifically, a pharmacist’s right to conscientiously object to PrEP prescribing may be influenced by the right for pharmacists to conscientiously object to providing any service as outlined within the Code of Ethics from the Nova Scotia College of Pharmacists.^20^ Finally, as discussed, increased workload during the COVID-19 pandemic and recent authorization of pharmacists in Nova Scotia to offer more services (e.g., prescribing for contraception, uncomplicated cystitis, shingles and Lyme disease) may have influenced respondents’ perceptions related to burden, opportunity costs and perceived effectiveness.

## Conclusion

This study found that community pharmacists in Nova Scotia had mixed reactions about accepting a PrEP prescribing service by pharmacists. Although some pharmacists expressed negative views towards PrEP prescribing, many believed it to be an important way to increase access to this critical intervention for underserved populations. Findings support future development of a PrEP prescribing service but not without important considerations for pharmacists’ workload, education and training and system factors relating to laboratory test ordering and reimbursement.■

## Supplemental Material

sj-pdf-1-cph-10.1177_17151635231152218 – Supplemental material for Community pharmacists’ acceptance of prescribing pre-exposure prophylaxis (PrEP) for human immunodeficiency virus (HIV) Click here for additional data file. Supplemental material, sj-pdf-1-cph-10.1177_17151635231152218 for Community pharmacists’ acceptance of prescribing pre-exposure prophylaxis (PrEP) for human immunodeficiency virus (HIV) by Connor Booker, Andrea L Murphy, Jennifer E. Isenor, Tasha D. Ramsey, Alesha J. Smith, Andrea Bishop, Deborah V. Kelly, Lisa Woodill, Greg Richard and Kyle John Wilby in Canadian Pharmacists Journal / Revue des Pharmaciens du Canada
