# Supplementary material for: Community pharmacists’ acceptance of prescribing pre-exposure prophylaxis (PrEP) for human immunodeficiency virus (HIV)
Source: Can Pharm J (Ott). 2023 Feb 28;156(3):137–49. doi: 10.1177/17151635231152218 (PMC10186867; doi:10.1177/17151635231152218)
Supplement: sj-pdf-1-cph-10.1177_17151635231152218 – Supplemental material for Community pharmacists’ acceptance of prescribing pre-exposure prophylaxis (PrEP) for human immunodeficiency virus (HIV) [file sj-pdf-1-cph-10.1177_17151635231152218.pdf]

APPENDIX 1 Interview guide for pharmacist interview sessions

Construct	Questions
Affective Attitude	In general, how do you feel about pharmacist prescribing of PrEP?
Burden	Will pharmacist prescribing of PrEP be a burden to you?<br><br>How do you see it affecting your current workload?
Ethicality	How do you think pharmacist prescribing of PrEP does or does not align with your personal values?
Intervention Coherence	Can you describe to me what a pharmacist prescribing program for PrEP would consist of?<br><br>What tasks do you think the pharmacist must complete in order to provide a PrEP prescribing service?
Opportunity Costs	If you were to offer a PrEP prescribing service, would you be concerned about having to give up something else?
Perceived Effectiveness	How effective do you think pharmacists will be at prescribing PrEP?<br><br>Do you have any hesitations or concerns in providing a PrEP prescribing service?
Self-Efficacy	Could you see yourself as a PrEP prescriber?<br><br>What barriers might you encounter when providing a PrEP prescribing service?

Booker C, et al. Community pharmacists' acceptance of prescribing for pre-exposure prophylaxis (PrEP) for human immunodeficiency virus (HIV). Can Pharm J (Ott) 2023;156. DOI: 10.1177/17151635221152218.
